# Work Motivation and Employment Outcomes in People with Severe Mental Illness

**DOI:** 10.1007/s10926-019-09839-0

**Published:** 2019-06-01

**Authors:** Miljana Vukadin, Frederieke G. Schaafsma, Sandra J. Vlaar, Jooske T. van Busschbach, Peter M. van de Ven, Harry W. C. Michon, Johannes R. Anema

**Affiliations:** 1 Department of Public and Occupational Health, Amsterdam Public Health Research Institute, Amsterdam UMC, Vrije Universiteit Amsterdam, Van der Boechorststraat 7, NL-1081 BT Amsterdam, The Netherlands; 2 Research Center for Insurance Medicine: collaboration between AMC– UMCG – UWV – VUmc, Amsterdam, The Netherlands; 3 University of Groningen, University Medical Center Groningen, Hanzeplein 1, 9713 GZ Groningen, The Netherlands; 4 Windesheim University of Applied Sciences, Campus 2, 8000 GB Zwolle, The Netherlands; 5 Department of Epidemiology and Biostatistics, Amsterdam Public Health Research Institute, Amsterdam UMC, Vrije Universiteit Amsterdam, Van der Boechorststraat 7, NL-1081 BT Amsterdam, The Netherlands; 6 Trimbos Institute, The Netherlands Institute of Mental Health and Addiction, Da Costakade 45, 3521 VS Utrecht, The Netherlands

**Keywords:** Severe mental illness, Work motivation, Vocational rehabilitation programs, Employment outcomes

## Abstract

*Purpose* To study associations between the level of self-reported work motivation and employment outcomes in people with severe mental illness (SMI) enrolled in a vocational rehabilitation program. *Methods* Data of 151 study participants, collected from a randomised controlled trial with a 30-month follow-up period, were used for a secondary data analysis. Multiple logistic regression, linear regression and cox regression analyses were performed to analyse the association between the level of work motivation at baseline and job obtainment, duration of job, and time until job obtainment during the 30-month follow-up period. *Results* No statistically significant associations were found between the level of work motivation and job obtainment (OR 1.83, 95% CI 0.55–6.06, p = 0.32), job duration (B = − 0.74, 95% CI − 2.37 to 0.89, p = 0.37, R-squared = 0.03), or time until job obtainment (HR = 1.53, 95% CI 0.64–3.68, p = 0.34). *Conclusions* The results of this study show no statistically significant associations between the level of work motivation and employment outcomes in people with SMI enrolled in a vocational rehabilitation program. These associations may be underestimated due to range restriction of the work motivation’s level. Further research is recommended to increase knowledge on the associations between work motivation and employment outcomes, as it could be relevant for further understanding success in vocational rehabilitation.

**Electronic supplementary material:**

The online version of this article (10.1007/s10926-019-09839-0) contains supplementary material, which is available to authorized users.

## Introduction

The unemployment rates for people with severe mental illness (SMI) are high [1–5], despite the fact they often do have a desire to work [6, 7]. There are many vocational rehabilitation approaches to help people with SMI to obtain and maintain employment, such as traditional vocational rehabilitation (TVR), in which a stepwise trajectory is offered with emphasis on assessment and matching procedures prior to job search [4, 5]. Another example is supported employment, focusing on a rapid search for competitive employment with ongoing support provided as long as needed to get and keep the job [8]. Several studies show that supported employment is more effective than other interventions in obtaining [4, 9, 10] and maintaining [9, 10] employment for people with SMI. Evidence also suggests that participants of supported employment programs need less time to find competitive employment in comparison with participants of TVR programs [4, 5].

The most widely used and researched model of supported employment is Individual Placement and Support (IPS). An important principle of IPS is that any person with SMI who expresses an explicit wish to work is eligible [11]. Motivation to work is important in most vocational rehabilitation programs, and is actually the only criterion for participation in IPS [11–13]. Expressing a wish to work, however, may not be fully the same as motivation to work, as the level of work motivation and the determinants involved can differ between people.

Motivation is a theoretical construct used to explain behaviour, such as behaviours linked to employment, and is included in several psychological theories [14–18]. The theory of planned behaviour [14], for example, suggests that the intention (including motivation) to obtain or maintain employment predicts the actual behaviour of obtaining or maintaining employment. This intention consists of three determinants: (1) attitudes (i.e. degree to which an individual has a favourable or unfavourable appraisal of obtaining or maintaining employment); (2) subjective norms (i.e. perceived social pressure to obtain or maintain employment); and (3) perceived behavioural control (i.e. self-efficacy; perceived ease or difficulty of obtaining or maintaining employment) [14].

Previous research on the association between these determinants of motivation and employment outcomes has also found that self-efficacy [19–21], attitudes and social pressure [19, 21] are indeed predictors of return to work in people on long-term sickness absence.

Research examining motivation in people with SMI, who expressed a wish to work and were enrolled in a vocational rehabilitation program, also supports the role of motivation as a predictor of employment outcomes [22–26]. Motivated people seem to link their motivation to a higher level of self-efficacy and control in their job search, a higher level of importance of work compared to other activities, and a higher level of social encouragement to find employment [24].

When not only people with an explicit wish to work, but all people with SMI who are enrolled in a vocational rehabilitation program are considered, the relation between expressing a wish to work or work motivation and employment outcomes becomes complex [12, 23, 27]. A study among people with SMI who expressed a wish to work has found a significant variability in the work motivation scores, and a positive relation between the level of work motivation and employment outcomes [23]. Other studies have also suggested that people who do not explicitly express interest in working have comparable employment outcomes to those who do express an initial interest [12, 27].

A better understanding of the complex relation between work motivation and employment outcomes in people with SMI who express a wish to work is important [11, 12], as it will help improve vocational rehabilitation outcomes. The aim of the present study was to study associations between the level of self-reported work motivation and (time until) job obtainment and job duration in people with SMI who were enrolled in IPS supported employment or a TVR program.

## Methods

### Study Design

Data collected from a Dutch randomised controlled trial (a study of cost-effectiveness of IPS on open employment in the Netherlands (SCION) [4]) were used for a secondary data analysis. The SCION study was registered in the Netherlands Trial Register (Trial ID NTR292; ISRCTN87339610), and approved by the National Medical Ethical Board in Mental Health (‘METIGG, kamer Zuid’, decision nr. 522) [4, 5].

### Sample, Setting, and Procedure

The SCION [4] study was conducted between 2005 and 2011, and designed as a multi-site randomised controlled trial, comparing IPS with TVR. Participants were recruited at four sites from regional community mental health care divisions targeted at adults with SMI. Inclusion criteria were: age between 18 and 65 years, explicit wish for competitive employment, and willingness to give informed consent. Exclusion criteria were: paid work at study entrance, full-time hospitalisation, engagement in another vocational rehabilitation program or another study with conflicting interest.

Data were prospectively collected during a 30-month follow-up period through self-report questionnaires and interviews with participants, complemented with information from vocational and mental health workers.

After baseline assessment, participants were allocated to IPS (n = 71) or TVR (n = 80). Randomization was performed by an independent agency and stratified by site and employment history (with or without some time in paid employment in the past 5 years).

### Measures

#### Competitive Employment and Employment Outcomes

In the SCION study [4], competitive employment was defined as having a paid job against prevailing wages, in a company or organization in the regular labour market, not set aside for persons with a disability, that is, in an integrated work setting. Information was derived from interviews with participants at baseline and after 6, 18 and 30 months, and from employment records filled out every 2 months by employment specialists. If no information was available from one or both of these two sources, the central case manager was interviewed by telephone for employment information.

In the current study, the employment outcomes were: (1) job obtainment, defined as having worked in a competitive job yes or no for one day or more; (2) job duration, measured as the total number of days worked in the first competitive job obtained; and (3) time until job obtainment, measured as the total number of days until first competitive job obtainment during the 30-month follow-up period.

#### Work Motivation

Work motivation was measured at baseline with a self-reported work motivation questionnaire [5], based on a questionnaire developed by Knispel and Schoemaker [28] for vocational rehabilitation clients. The aim of the original questionnaire was to understand the determinants of work motivation, by exploring clients’ ideas about competitive employment. This questionnaire was inspired by the aforementioned psychological theories [14–18], and contained 101 items. For the SCION study [5], the original questionnaire was adjusted by the SCION research team, by selecting 27 from the 101 items. The 27 items were rated on a four-point Likert scale; answer categories were: strongly agree (1), agree (2), disagree (3) and strongly disagree [4]; ‘not applicable’ was also an option for items regarding social pressure.

Internal consistency (Cronbach’s α) of the adjusted work motivation questionnaire and four subscales was evaluated in the study sample of 151 participants at baseline. The four subscales were: self-consciousness regarding work, drive to work, seeing opportunities and action readiness. The total work motivation scale of 27 items had good internal consistency (α = 0.82). The internal consistency of the total scale could only be assessed in a small subsample of 27 patients due to a considerable amount of missing data or the answer ‘not applicable’ on the five items regarding social pressure. When the item that was most frequently missing (i.e. ‘For my partner, it is important that I work’) or all five items regarding social pressure were omitted, the total work motivation scale still showed acceptable internal consistency (resp. α = 0.78 in a subsample of 103 participants, and α = 0.76 in a subsample of 134 participants). Cronbach’s α showed good internal consistency for the subscale self-consciousness regarding work (e.g., ‘I know what type of work I want to do’) (α = 0.82 in a subsample of 150 participants), questionable internal consistency for the subscale drive to work (e.g. ‘It is very important for me to start working again’) (α = 0.65 in a subsample of 149 participants) and poor internal consistency for the subscales seeing opportunities (e.g. ‘I think I have a good chance to start working again’) and action readiness (e.g. ‘I am willing to do a short course or training to increase my chances of finding a job’) (resp., α = 0.58 in a subsample of 140 participants and α = 0.57 in a subsample of 146 participants).

In the present study, the baseline work motivation and the subscale self-consciousness regarding work (i.e. subscale showing good consistency) score served as independent variables. The mean of all non-missing items of the adjusted work motivation questionnaire was used, where it was required that the participants completed at least 80% of the items; participants with more than 20% of items missing were excluded from the analyses. The subscale self-consciousness regarding work score was based on the mean of 4 of the 27 items. Online Resource 1 provides the adjusted work motivation questionnaire and an overview of the internal consistency for the total scale and the subscales of this questionnaire, including corresponding items.

#### Covariates

The following covariates were considered as candidate confounders for the relation between work motivation and employment outcomes: gender, age, education, work history, clinical diagnosis (yes/no psychotic disorder; derived from mental health care professionals), self-esteem (RSE [29]), mental health (MHI-5 [30]), and vocational rehabilitation program (IPS/TVR). Candidate confounders were chosen based on the literature [4, 31–33].

### Statistical Analyses

To evaluate whether work motivation was associated with job obtainment, logistic regression analysis was used with job obtainment as the dependent and work motivation as the independent variable. Association between the participants’ score on the self-consciousness regarding work subscale and job obtainment was assessed in a similar way.

To evaluate whether work motivation was associated with job duration, linear regression analysis was used with total number of days worked as the dependent variable. This analysis was based on participants that obtained employment within 30 months; participants who did not start a competitive job or for whom specific data on number of days in employment was missing, were excluded from this analysis. Association between the participants’ score on the self-consciousness regarding work subscale and job duration was assessed in a similar way. Because job duration was skewed to the right, a log transformation was used before analysis.

To evaluate whether work motivation was associated with time (total number of days) until job obtainment, Cox regression analysis was used, where the event was defined as starting a competitive job and maintaining it for at least 1 day. Participants who did not start a competitive job within the 30-month follow-up period were censored at the end of the follow-up period. Participants who were lost to follow-up before starting a competitive job, were censored at the end of the period over which accurate information was available. Association between the participants’ score on the self-consciousness regarding work subscale and time until job obtainment was assessed in a similar way.

For all research questions, both a crude (adjusted for vocational rehabilitation program only) and an adjusted analysis (adjusted for all predefined confounders) were performed. For all analyses, a two-sided significance level of 5% was used and 95%-confidence intervals (CIs) for odds ratios (ORs), regression coefficients and hazard ratios (HRs) were calculated. All statistical analyses were performed using SPSS 22.0 (SPSS, Chicago, IL, USA).

## Results

### Baseline Characteristics and Employment Outcomes

A total of 151 participants were included in this study. The baseline characteristics and employment outcomes of the participants who did not obtain competitive employment (n = 100) and who did obtain competitive employment (n = 51) within the 30-month follow-up period are shown in Table 1. The mean work motivation score was 2.9 with a standard deviation of 0.3, in a subsample of 149 participants. A total of 71 participants (47%) were enrolled in IPS and 44 of the 51 participants with competitive employment (86%) obtained the job within 18 months. The median number of days until competitive job obtainment was 198, and the median of the number of days worked in the first, competitive job was 138.

**Table 1:** Baseline characteristics and employment outcomes of the participants within the 30-month follow-up period

	All participants (n = 151)	Participants without a competitive job (n = 100)	Participants with a competitive job (n = 51)
Socio-demographic characteristics			
Gender male (%)	112 (74)	78 (78)	34 (67)
Mean age in years (SD)	34.9 (10.5)	35.7 (10.2)	33.4 (10.9)
Married (%)	13 (9)	7 (7)	6 (12)
Low and medium level of education (%)	130 (87)	85 (85)	45 (88)
Employment in past 5 years (%)	92 (61)	55 (55)	37 (73)
Worked competitively in past 5 years (%)	79 (86)	44 (44)	35 (69)
Disability benefits (%)	81 (60)	58 (58)	23 (45)
Clinical characteristics			
Admission to mental hospital in past 6 months (%)	38 (34)	23 (23)	15 (29)
Psychotic disorders (%)	90 (63)	59 (59)	31 (61)
Self-report measures			
Mean work motivation score (SD), range 1–4	2.9 (0.3)	2.8 (0.3)	2.9 (0.2)
Mean score RSE (self-esteem) (SD), range 0–3	1.8 (0.5)	1.8 (0.5)	1.9 (0.5)
Mean score MHI-5 (mental health) (SD), range 0–100	59.9 (18.7)	59.4 (18.9)	60.9 (18.4)
Vocational rehabilitation program			
Individual placement and support (%)	71 (47)	40 (40)	31 (61)
Employment outcomes			
Found competitive employment between baseline and 18 months (%)	44 (29)	0 (0)	44 (86)
Found competitive employment between 18 and 30 months (%)	7 (5)	0 (0)	7 (14)
Median of number of days until job obtainment [IQR]^a^			198.0 [107.0–455.0]
Median of number of days worked in first job [IQR]^b^			138.0 [61.0–302.5]

^a^Subsample of people competitively employed, n = 51

^b^Subsample of people competitively employed, n = 49

### Relation Between Work Motivation and the Employment Outcomes

The logistic regression analysis on job obtainment was based on 149 participants; two participants who had filled in less than 80% of the items of the work motivation questionnaire were excluded from analyses. No statistically significant association was found between work motivation and job obtainment (OR 1.83, 95% CI 0.55–6.06, p = 0.32). This association remained non-significant after adjustment for all covariates. There was also no significant association between the self-consciousness regarding work score and job obtainment (OR 0.99, 95% CI 0.55–6.06, p = 0.32).

The linear regression analysis on job duration was based on 49 participants who had obtained employment within 30 months; two participants were excluded from the analyses due to missing specific data on number of days in employment. No statistically significant association was found between work motivation and time in the first job obtained (B = − 0.74, 95% CI − 2.37 to 0.89, p = 0.37, R-squared = 0.03). This association remained non-significant after adjustment for all covariates. There was also no significant association between the self-consciousness regarding work score and time in the first job obtained (B = − 0.37, 95% CI − 1.08 to 0.34, p = 0.30, R-squared = 0.04); the association remained non-significant after adjustment.

The Cox-regression analysis on the time until job obtainment was based on 148 participants; one participant had missing data regarding both employment and follow-up period, and two participants had filled in less than 80% of the items of the work motivation questionnaire. All three were excluded from the analyses. No statistically significant association was found between work motivation and the time until job obtainment (HR = 1.53, 95% CI 0.64–3.68, p = 0.34). This association remained non-significant after adjustment for all covariates. There was also no significant association between the self-consciousness regarding work score and the time until job obtainment (HR = 0.98, 95% CI 0.63–1.52, p = 0.91); the adjusted HR remained non-significant.

## Discussion

The purpose of this study was to study associations between the level of self-reported work motivation at baseline and (the time until) job obtainment and job duration during a 30-month follow-up period in people with SMI who had expressed a wish to work and were enrolled in a vocational rehabilitation program. The results of this study showed no statistically significant associations between the level of work motivation at baseline and the employment outcomes, independent of vocational program type.

### Comparison with Other Studies

In contrast to previous research examining motivation in people with SMI enrolled in vocational rehabilitation programs [22–26], the present study did not find a significant association between the level of work motivation at baseline and employment outcomes. The present study, however, assessed other determinants of motivation, used a different assessment for motivation and employment outcomes, and had a much longer follow-up period. Differences in both labour market dynamics and welfare systems may also have played a role, as this study was conducted in the Netherlands, whereas previous studies were conducted outside of Europe. The labour market in the Netherlands is characterized by restrictive regulations regarding temporary employment and relatively high minimum wages. The Netherlands also has a generous welfare system, which seems to be associated with the so-called ‘benefit trap’ (financial disincentives to return to work); this ‘benefit trap’ seems to be an impediment to successful vocational rehabilitation [34]. All these differences make it difficult to compare the results of the present study with previous research. In the present study, a limited variability in the work motivation scores was found. One explanation could be that the explicit wish to work was one of the inclusion criteria in the Scion study [4], as this is the only criterion for participation in IPS [11]. Another explanation could be that participants who were less motivated dropped out before entering the study, as participants were interviewed several times during the 30-month follow-up period and had to consent to research procedures such as the randomisation [4]. In contrast to this finding, Reddy et al. [23] did find a significant variability in the work motivation scores in a comparable study sample of people with SMI who had also expressed a wish to work. This limited variability in the work motivation score may also explain not finding a significant association between the level of work motivation at baseline and employment outcomes in this study. Another explanation could be the small sample size and the small number of participants that had obtained competitive employment within 30 months (only 34%).

### Strengths and Limitations

This is the first study in Europe on the complex relation between work motivation and employment outcomes in people with SMI who are enrolled in a vocational rehabilitation program. One of its strengths is the long follow-up period in comparison to other studies on motivation and employment outcomes in people with SMI [22–26]. The use of a work motivation questionnaire, inspired by several theoretical frameworks [14–18], is also a strength. The main limitation of this study is that there may be a selection bias of highly motivated participants in the SCION study [4], as one of the inclusion criteria was an explicit wish for competitive employment. Therefore the association between work motivation and employment outcomes may be underestimated due to range restriction of the work motivation’s level. The work motivation questionnaire was originally not designed and validated for people with SMI [28]. Furthermore, a considerable amount of data was missing on five items of this questionnaire regarding social pressure. Social pressure, however, is an important factor to take into consideration when exploring grounds for motivation to obtain and maintain employment [32]. Missing items were replaced by the mean score of the participants’ score on the completed items, assuming that responses on the completed items are representative for the items regarding social pressure. This may not be the case in general or more specific for people with SMI. Although internal consistency for the total work motivation scale was adequate, it was not sufficient for most of the subscales. Analyses were therefore only performed for the total scale and the subscale self-consciousness regarding work. Another limitation may be the small sample size; of the total sample only 34% of the participants (n = 51) obtained employment within 30 months. The Scion study [4], which was used for this secondary data analysis, was not powered to answer the research questions of the present study. Furthermore, the data used for the present study might be outdated, as these data were collected for another study [4, 5], conducted between 2005 and 2011, a period in which the labour market situation fluctuated due to the 2008 financial crisis. Although this may have influenced employment outcomes, it is uncertain whether this would also have influenced the relation between work motivation and employment outcomes. In addition, the analyses for job duration were restricted to participants which worked in a competitive job for at least 1 day, which limits generalizability to the whole group of people with SMI.

### Implications for Research and Practice

Policy makers and professionals in mental health care and vocational rehabilitation have been increasingly investing a considerable amount of time and funding in helping people with SMI to obtain and maintain competitive employment, by aiming to improve implementation of vocational rehabilitation programs [35–37]. Therefore, it is important to conduct more research on potentially changeable predictors of employment outcomes in this population, such as work motivation in the present study. Besides of striving for a sufficient sample size, future research should develop and validate a questionnaire on motivation to workfor people with SMI and based on a theoretical framework, such as the theory of planned behaviour [14]. Work motivation and employment outcomes should also be assessed at multiple time points, as work motivation seems to be a dynamic concept that can increase over time [22, 38]. Understanding the relation between work motivation, including its factors of influence [14], and employment outcomes in people with SMI who have expressed a wish to work, may help improve vocational rehabilitation outcomes. Additional interventions or training, for example social skills training or an extra course to improve knowledge for a specific job, can be used to improve potentially changeable components of work motivation, such as self-efficacy. Such integration of vocational rehabilitation with additional interventions is more effective with regard to employment outcomes for people with SMI, than a vocational rehabilitation programme alone [10].

## Conclusion

The results of this study show no statistically significant associations between the level of work motivation and employment outcomes in people with SMI enrolled in a vocational rehabilitation program. These associations may be underestimated due to range restriction of the work motivation’s level. Further research is recommended to increase knowledge on the associations between work motivation and employment outcomes, as it could be relevant for further understanding success in vocational rehabilitation.

## Electronic supplementary material

Below is the link to the electronic supplementary material.

Supplementary material 1 Online Resource 1: Work motivation and employment outcomes in people with severe mental illness. Work motivation questionnaire and an overview of internal consistency for total scale and subscales. (DOCX 20 kb)
