# Supplementary material for: Work Motivation and Employment Outcomes in People with Severe Mental Illness
Source: J Occup Rehabil. 2019 Jun 1;29(4):803–9. doi: 10.1007/s10926-019-09839-0 (PMC6838012; doi:10.1007/s10926-019-09839-0)
Supplement: Supplementary file 1 — Supplementary material 1 Online Resource 1: Work motivation and employment outcomes in people with severe mental illness. Work motivation questionnaire and an overview of internal consistency for total scale and subscales. (DOCX 20 kb) [file 10926_2019_9839_MOESM1_ESM.docx]

**Work motivation and employment outcomes in people with severe mental illness**

**Journal of Occupational Rehabilitation**

Miljana Vukadin, M.D., Frederieke G. Schaafsma, M.D., Ph.D., Sandra J. Vlaar, M.D., Jooske T. van Busschbach, Ph.D., Peter M. van de Ven, Ph.D., Harry W.C. Michon, Ph.D., Johannes R. Anema, M.D., Ph.D.

Corresponding author:

Miljana Vukadin

Amsterdam UMC, Vrije Universiteit Amsterdam, Department of Public and Occupational Health, Amsterdam Public Health research institute, Van der Boechorststraat 7, 1081 BT Amsterdam, The Netherlands

Research Center for Insurance Medicine: collaboration between AMC– UMCG – UWV – VUmc, Amsterdam, The Netherlands

E: [m.vukadin@vumc.nl](mailto:m.vukadin@vumc.nl)

**Online Resource 1.**

**Work motivation questionnaire**

The following statements concern your ideas about regular paid work and getting back to work. For each statement, please indicate to which extent you agree or disagree with the statement. Point out the answer that fits best.

- Strongly agree (1)
- Agree (2)
- Disagree (3)
- Strongly disagree (4)
- Not applicable (only an option for item 18, 19, 20, 21)

1. It is very important for me to start working again.
2. I am fine with not having a job.
3. Work is important in my life.
4. Without work, I can also live a happy life.
5. If I would win the lottery, I would never work another day.
6. I am willing to change the way I organize my private life, if that would be necessary to get a job.
7. I am willing to do a short course or training to increase my chances of finding a job.
8. I am willing to move to another city for a job.
9. I do not mind working irregular hours.
10. I don’t have much time to apply for a job right now.
11. I do not plan on constantly spending time searching for a job.
12. I have no clue as to what jobs suit me.
13. I have barely spent time thinking about what types of jobs I could do in the future.
14. I have some ideas about what jobs would suit me, but I have to investigate this further.
15. I know what type of work I want to do (for example: administrative, care etc.).
16. I have spoken to friends about my plans to start working again.
17. I can make clear to others what type of job I want to do.
18. For my family members, it is important that I work.
19. For my friends, it is important that I work.
20. For my mental health care providers (case manager, psychologist, psychiatrist), it is important that I work.
21. For my partner, it is important that I work.
22. I think that finding a job is a challenge.
23. I think I have a good chance to start working again.
24. Given my health issues, it will be very difficult for me to start working again.
25. With my health issues, eventually it will be possible to start working again.
26. I think there are barely jobs that I can do.
27. Employers are not interested in me.

**Internal consistency of the work motivation questionnaire**

Cronbach’s alpha’s for the total scale and the subscales

|  | Cr. alpha | Items | Number of participants |
| --- | --- | --- | --- |
| Total Scale | 0.82 | All | 27 |
|  | 0.78 | All, excluding 21* | 103 |
|  | 0.76 | All, excluding 16, 18, 19, 20, 21^*^ | 134 |
| Subscales |  |  |  |
| Self-consciousness regarding work | 0.82 | 12, 13, 15, 17 | 150 |
| Drive to work | 0.65 | 1, 2, 3, 6, 22 | 149 |
| Seeing opportunities | 0.58 | 23, 24, 25, 26, 27 | 140 |
| Action readiness | 0.57 | 4, 7, 8, 9, 10, 11, 14 | 146 |

^*^Item(s) regarding social pressure
